# The genetically programmed rhythmic alteration of diurnal gene expression in the aged *Arabidopsis* leaves

**DOI:** 10.3389/fpls.2024.1481682

**Published:** 2024-11-04

**Authors:** Sukjoon Jung, Hyunmin Kim, Juhyeon Lee, Myeong Hoon Kang, Jungyeon Kim, Jong Kyoung Kim, Pyung Ok Lim, Hong Gil Nam

**Affiliations:** ^1^ Department of New Biology, Daegu Gyeongbuk Institute of Science&Technology (DGIST), Daegu, Republic of Korea; ^2^ Center for Plant Aging Research, Institute for Basic Science, Daegu, Republic of Korea; ^3^ Department of Life Sciences, Pohang University of Science and Technology (POSTECH), Pohang, Republic of Korea; ^4^ Ever Summer Labs for Aging Research, Daegu Catholic University, Gyungsan, Republic of Korea

**Keywords:** circadian clock, aging, diurnal expression, transcriptomics, plant gene regulation

## Abstract

The circadian clock regulates the daily pattern of temporal gene expression. In Arabidopsis, aging is associated with a shortening of the endogenous period of circadian rhythms under circadian conditions. However, the functional link between the circadian clock and aging under diurnal conditions and its physiological relevance remain elusive. In this study, we investigate and characterize the effect of aging on the waveforms of rhythmic gene expression patterns under light/dark cycles. Our analysis revealed that the diurnal rhythmic patterns of core clock genes undergo significant rhythmic alteration with phase shift and change of waveforms in aged plants compared to younger plants. Transcriptomic analysis indicated that this age-dependent rhythmic alteration occurs not only in core clock genes but also globally. Due to the rhythmic alteration patterns of the diurnal rhythmic gene expression, aged plants experience subjectively a shorter day and longer night. We also observed that genetic mutants of core clock component genes exhibited broadly yet distinctively altered changes in diurnal rhythmic gene expression patterns as aging progresses. Collectively, our findings support that age-dependent rhythmic alteration of diurnal gene expression rhythms reprograms the timetable of daily gene expression, leading to the physiological changes required for plant senescence.

## 1 Introduction

Most, if not all, living organisms undergo age-dependent physiological changes that eventually lead to age-associated disintegration or senescence and death at both the cellular and organismal levels. Life spans are species-specific, with each species exhibiting distinctive patterns in their aging and death processes in their given environmental settings (Jones et al., 2014). Thus, aging has evolved as a life history strategy to maximize overall fitness (Gaillard and Lemaitre, 2017), as well exemplified in plants.

Plants exhibit diverse life history traits (Adler et al., 2014). As the primary producers in many ecosystems, plants rely on leaves, the major site of photosynthesis, which provide the fundamental basis of their survival. Throughout their lifespan, leaves undergo a series of developmental and physiological shifts accompanied by orderly changes in metabolism and gene expression (Buchanan-Wollaston et al., 2003). Leaf aging is a crucial developmental strategy in both annual and perennial plants, as the cellular materials accumulated during the leaf’s growth stage are converted into exportable nutrients for relocation to newly developing or reproductive organs, or storage in roots or stems (Lim et al., 2007). Therefore, plants must precisely time the onset and progression of leaf senescence in each ecological setting to ensure optimal production of offspring and overall plant survival (Sade et al., 2018; Woo et al., 2019). However, the mechanisms by which plants perceive and process time information, and thus control aging are not fully understood, despite aging being a widely occurring biological phenomenon.

The circadian clock is a biological system that regulates daily timekeeping, consisting of interlocked feedback loops of gene expression that drive physiological rhythms. This system phases and sequences biological events to ensure they occur in a coordinated manner at the optimal times of the day (Dunlap, 1999). Additionally, the circadian clock enables organisms to anticipate predictable environmental changes and adjust their developmental and physiological responses accordingly (Zheng and Sehgal, 2008). The clock system is plastic, continuously rewiring through its interplay with biological processes, including metabolism, hormone signaling, and stress pathways, as well as with environmental factors such as light and temperature. This intrinsic plasticity suggests that the clock is not merely a timekeeper but a complex developmental regulator (Oakenfull and Davis, 2017). In plants, many aging-related physiological processes, including growth, development, and flowering, are regulated by the circadian clock (Sanchez and Kay, 2016).

An intimate interaction between aging and the circadian clock is also observed in animals. With increasing age, the circadian system undergoes significant changes in amplitude and period, affecting physiological rhythms (Hood and Amir, 2017). Aging reprograms the circadian transcriptome in a highly tissue-specific manner (Sato et al., 2017; Solanas et al., 2017), and disruption of the circadian clock affects the aging process (Kondratov et al., 2006; Yu and Weaver, 2011). In *Arabidopsis thaliana* (hereafter, Arabidopsis), the free-running period of circadian rhythms (i.e., under constant light (LL) or constant dark (DD), so that not influenced by environmental transitions) is influenced by leaf age, becoming shorter in older leaves (Kim et al., 2016). However, mutations in the gene, *TIMING OF CAB EXPRESSION 1 (TOC1)*, which encodes a core clock component, abolish age-dependent changes in the period, providing the first insight into how age-dependent changes in the circadian clock are incorporated into developmental decisions (Kim et al., 2016). Furthermore, disruption of other core clock components leads to delayed or earlier senescence, suggesting that the circadian clock and leaf aging are intimately interlinked (Kim et al., 2018a). Nevertheless, how the circadian clock is reprogrammed by aging to modulate age-dependent leaf physiology remains largely unknown. This is partly because circadian rhythms in plants are often monitored in LL or DD which does not reflect the daily environmental changes that plants experience in nature.

We hypothesized that leaf aging uniquely influences daily clock activities under diurnal conditions compared to circadian conditions. To address this, we took an integrated approach combining genetics and transcriptomics to dissect the effects of aging under both diurnal and circadian conditions. Our analysis revealed age-dependent changes of gene expression patterns, or “rhythmic alteration” with phase shift and change of waveforms, in diurnal conditions, which distinctive from one in circadian conditions. The time-course transcriptomic study showed that the rhythmic alteration rhythmic alteration effect extends beyond a few clock marker genes, impacting the transcriptome and significant biological processes. We further validated these aging-induced rhythmic alteration rhythmic alteration effects in clock mutants. Our findings highlight the widespread impact of aging on circadian clock activities and its functional relevance to plant physiology.

## 2 Materials and methods

### 2.1 Plant materials and growth conditions

*Arabidopsis thaliana* (hereafter, Arabidopsis) Col-0 was used as the wild-type background in gene expression experiments. Circadian clock activity in Arabidopsis was monitored using transgenic lines expressing firefly *luciferase* under the control of the *CCR2* (*pCCR2::LUC*) or *CCA1* (*pCCA1::LUC*) promoters in wild-type plants and in *prr9-1*, *toc1-101*, *elf3-7*, *prr7-3*, and *elf4-209* mutants, as described previously (Kim et al., 2018a).

Plants were grown in a growth chamber at 22°C under a 16 h light/8 h dark cycle (long day; LD 16:8) with a light intensity of 100 μmol m^−2^ s^−1^ white light. Plants were transferred to the chamber with the same light intensity to measure luciferase emissions from transgenic leaves.

### 2.2 Luciferase assay

Luminescence was measured in leaves from transgenic plants harboring *luciferase* reporters, as described above. The third rosette leaves were excised from transgenic plants at given ages and transferred to 8-, 24-, or 48-well microplates containing 1 mM luciferin (SYNCHEM, Felsberg/Altenburg, Germany). Luminescence signals were captured for 10 minutes every 20 minutes for 4 days using a CCD camera, and luminescence intensities from each pixel were stacked (**Supplementary Figure 1A**). The stacked images were segmented and processed using a bespoke program we developed. The scripts used in the analyses is available on GitHub (https://github.com/jsjoon0719/Daily-timetable-of-diurnal-gene-expression-rhythms-undergoes-a-programmed-rhythmicalteration-toward-night-patte/tree/main).

### 2.3 Physiological time

We generated a predictive model using the 21 DAS plant as a reference and calculated physiological time for an arbitrary transcriptome profile. For learning, we used the ridge regressor function of the SciPy package with the default value of parameters (Virtanen et al., 2020). The predictive model consisted of two predictors: one predicts cos(2π*sampling time/24h) and the other predicts sin(2π*sampling time/24h). By calculating the angle between the starting line and the point (output1, output2), the model predicts the sampling time of a given transcriptome. This triangular regression was used to calculate the error function between daily times. The code used for analyses is available on GitHub (https://github.com/jsjoon0719/Daily-timetable-of-diurnal-gene-expression-rhythms-undergoes-a-programmed-rhythmicalteration-toward-night-patte/tree/main).

We performed a cross-validation analysis to validate the model’s ability to parameterize the physiological time of the transcriptomes. To verify the reliability of the physiological time produced by the model, we created a model based on data from which one of the samples (collected at ZT1, ZT5, ZT9, ZT13, ZT17, and ZT21) had been removed. We used the sample time that was not utilized in training as an input to predict the sampling time. The predicted time showed less than 2 hours of error compared with the actual sampling time.

### 2.4 Calculation of full width at half maximum

To calculate the full width at half maximum (FWHM) value, we assumed that the cycling curve increased monotonically on the left side and decreased monotonically on the right side around a single maximum point. The scale was normalized so that the minimum value became 0 and the maximum value became 1, and the value obtained by substituting 0.5 into the inverse of the expression function F concerning time for continuous-time values ​​T1 and T2 where the expression value was greater than or equal to 0.5 was calculated using Equation 1. FWHM was the difference between the two F^-1^(0.5). The code used for analyses is available on GitHub:(https://github.com/jsjoon0719/Daily-timetable-of-diurnal-gene-expression-rhythms-undergoes-a-programmed-rhythmicalteration-toward-night-patte/tree/main).


(1) $${\text{F}}^{-1}\left(0.5\right)=\text{T}2\frac{\text{F}\left(\text{T}1\right)-0.5}{\text{F}\left(\text{T}1\right)-\text{F}\left(\text{T}2\right)}+\text{T}1\frac{\text{F}\left(\text{T}2\right)-0.5}{\text{F}\left(\text{T}1\right)-\text{F}\left(\text{T}2\right)}$$

### 2.5 Gene expression analyses

RT-qPCR was done as described. In order to extract total RNA, 21 DAS and 35 DAS *Arabidopsis* leaves were sampled for 24 hours at 30-minute intervals. For visualizing oscillation pattern, we extended 12 hours using initial 12 hours’ gene expression again. Each pooled *Arabidopsis* leaves sample was frozen with liquid nitrogen. Total RNA was extracted from the leaves using Qiazol lysis reagent (QIAGEN, Valencia, CA). To remove DNA contamination, DNase I (Ambion, Austin, TX, USA) was treated on each RNA sample. For each sample, 750 ng of total RNA was reverse transcribed using ImProm II reverse transcriptase (Promega, Madison, WI, USA). qRT-PCR analysis was carried out by using iTaq Universal SYBR Green Supermix (Bio-Rad, Hercules, CA, USA) and CFX96 Touch™ Real-time PCR Detection System (Bio-Rad, Hercules, CA, USA). *ACT2* (*AT3G18780*) was used as an internal control. The relative expression represents means of 2(−ΔCT) from biological replicates, in which ΔCT = (CT of Gene of Interest – CT of internal control) (Livak and Schmittgen, 2001). The primers used are listed in supplemental dataset (**Supplementary Data Sheet 7**).

### 2.6 RNA sequencing analysis

Total RNA isolated from third and fourth rosette leaves at ZT1, ZT5, ZT9, ZT13, ZT17, and ZT21 from 21 DAS or 35 DAS aged Col-0 plants was used to prepare RNA-seq libraries with TruSeq™ RNA Library Preparation Kit (Illumina, CA, USA). Library construction and sequencing were carried out by THERAGEN BIO (South Korea) with Illumina NovaSeq6000 (Illumina, CA, USA). Filtered reads were aligned to the *Arabidopsis thaliana* genome (TAIR10) (Huala et al., 2001) using the aligner STAR v.2.3.0e with the default option (Dobin et al., 2013). The tool htseq-count of Python package HTSeq was used to count reads mapped to annotated genes (Anders et al., 2015). Trimmed mean of M-values (TMM) algorithm was used to normalize raw read counts using edgeR (v3.12.1) (Robinson et al., 2010).

### 2.7 Selecting genes with oscillating expression patterns

To select genes with daily oscillations, we used rhythmicity detection analysis through cosine curve fitting provided by CosinorPy (Moskon, 2020). We performed cosinor regression of the data to the rhythmic function of a 24 h period and calculated the significance of the model. We chose 0.0005 as the threshold for oscillating genes. The code used for analyses is available on GitHub (https://github.com/jsjoon0719/Daily-timetable-of-diurnal-gene-expression-rhythms-undergoes-a-programmed-rhythmic-alteration-toward-night-patte/tree/main).

We performed a permutation test to select genes with a single FWHM. A curve was created by randomly selecting one replicate of the measured values ​​that existed for each time-point, and we then determined whether this curve had a single FWHM. This operation was repeated 10,000 times; a gene with a single FWHM ratio of > 0.95 was defined as a gene with a single FWHM. The generated curves were used for a statistical analysis of the mean expression and length of FWHM. The code used for analyses is available on GitHub: (https://github.com/jsjoon0719/Daily-timetable-of-diurnal-gene-expression-rhythms-undergoes-a-programmed-rhythmic-alteration.toward-night-patte/tree/main).

### 2.8 Statistical analysis

For different group analyses, we used (one-way or two-way) ANOVA and Dunnett’s multiple comparison test. Two-tailed t-test were used for measuring the difference between two groups. All statistical analyses were indicated in the Figure legends. Independent experiments were performed at least three times, unless indicated otherwise. Statistical analyses were performed using Excel and SciPy.

## 3 Results

### 3.1 Free-running circadian rhythms undergo age-dependent rhythmic alteration

**Figure 1:**
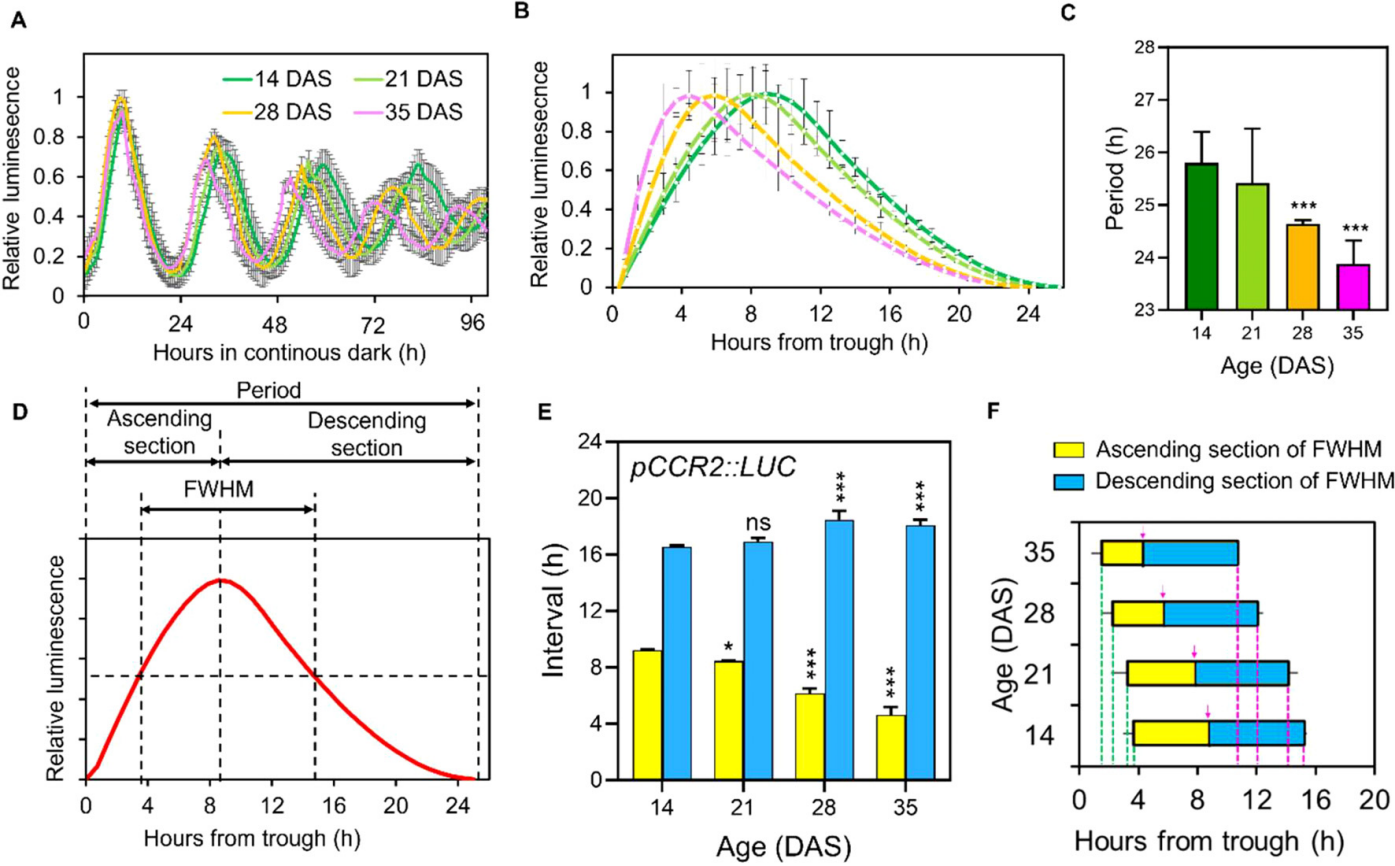
Age-dependent rhythmic alteration of the free-running circadian rhythm of pCCR2::LUC expression. **(A)** Total luminescence intensity(relative intensity ± SD) from leaves ofplants expressing pCCR2::LUC in DD at the indicated ages after entrainment to LD cycles (n = 16 leaves for each condition). **(B)** Normalized and detrended circadian oscillatory curves of the first three cycles of pCCR2:LUC expression (relative intensity ± SD) at each age. **(C)** Circadian periods (hours ± SD) were measured from the data in (B); *** p-value < 0.001, using one-way ANOVA and Dunnett’s multiple comparison test (n = 12 leaves). **(D)** Parametrization of the waveform of the circadian oscillation curves. The curves were divided into ascending and descending sections to parameterize the age-dependent changes in the time from trough to crest and from crest to trough, respectively. FWHM value denotes the time elapsed (duration) between the ascending and descending half maximum points. **(E)** Age-dependent changes of the ascending (yellow bars) and descending sections (blue bars) (hours ± SD); *p-value < 0.05 and ***p-value < 0.001 using two-way ANOVA and Dunnett’s multiple comparison test (n = 3 biological replicates) against the period of 14 DASsamples. **(F)** Age-dependent changes in FWHM values (hours ± SD). The ascending (yellow bars) and descending parts (blue bars) were divided by the crest timepoints indicated by magenta arrows in each age. Ascending of half maximum is from magenta dashed line to magenta arrow and descending of half maximum is from magenta arrow to green dashed line. ns, not significant.

In a previous study (Kim et al., 2016), we reported age-dependent shortening of circadian rhythms in the expression of circadian clock marker genes, *COLD CIRCADIAN RHYTHM AND RNA BINDING 2* (*CCR2*) and *CIRCADIAN CLOCK ASSOCIATED 1* (*CCA1*) in Arabidopsis under LL. *CCR2* is a clock output with an evening expression peak and *CCA1* is a core clock oscillator with a morning peak (Huang et al., 2012). In this study, we re-examined the promoter activity of *CCR2* driving the *LUCIFERASE* gene (*pCCR2*:*LUC*) across various aging stages (14, 21, 28, and 35 days after sowing (DAS)) in Arabidopsis Col-0 under DD at high time resolution (~20 min intervals) following the long-day (LD; 16h light/8h dark) entrainment using a high-resolution CCD camera system (**Figure 1A**; **Supplementary Figure 1A**). This analysis revealed an age-dependent shortening of the free-running circadian period as leaves aged: 25.7 hours at 14 DAS; 25.4 hours at 21 DAS; 24.5 hours at 28 DAS; 23.9 hours at 35 DAS (**Figures 1B, C**), consistent with the previous findings (Kim et al., 2016). In addition to the period shortening, we observed age-related changes in rhythmicity, specifically a skewing of the *pCCR2*::*LUC* rhythm towards the evening as leaves aged (**Figures 1B, C**). After normalizing the expression levels to the maximal values across time-points (**Supplementary Figures 1B–D**), we noted shifts in both the phase of the peak and the shape of the circadian waveforms. We refer to this phenomenon as ‘rhythmic alteration’ throughout this study. Although ‘rhythmic alteration’ is traditionally a mathematical term describing the smooth transformation or distortion of a space, shape or function (Milliron et al., 2002), it has recently been applied to describe altered rhythmic oscillations in circadian regulation (van Bree et al., 2022; Williams et al., 2020).

To comprehensively quantify the level of rhythmic alteration observed in the *pCCR2*::*LUC* rhythm, we applied Full Width at Half Maximum (FWHM) analysis (Fukami et al., 2017), a statistical method to describe the width of curves, to the circadian curves of *pCCR2*::*LUC*. In this analysis, rhythmic alteration patterns of the circadian curves were parameterized by binary sectioning dividing a single cycling curve into ascending and descending sections using the peak time-point (crest) as a reference point (**Figure 1D**). The FWHM of a circadian curve is defined as the interval between the ascending and the descending half maximum points (**Figure 1D**), representing the “duration” and “timing” of gene expression higher than half of its maximum level during a daily cycle. Interestingly, the analysis revealed: (1) the ascending section of the circadian curve of *pCCR2*::*LUC* decreased, while the descending section increased, as leaves aged; (2) the change in the ascending section (-37.5%, from 9 hours at 14 DAS to 5 hours at 35 DAS) was greater than that in the descending section (+12.5%, 16 hours at 14 DAS to 18 hours at 35 DAS); and (3) the ascending and the descending half maximum points, as well as peak point, advanced in aged plants (**Figures 1E, F**). Collectively, the analysis showed that the lengths and timing of the FWHM in the circadian rhythms of *pCCR2*::*LUC* decreased and shifted, respectively, with leaf age, demonstrating that the pattern of the cycling curve of endogenous circadian rhythm underwent age-dependent rhythmic alteration.

### 3.2 Distinct patterns of age-dependent rhythmic alteration of clock gene expression under diurnal conditions

We next investigated whether the age-dependent of gene expression rhythms under circadian conditions also occurs under diurnal cycles. The internal circadian rhythm interacts with the environmental cycle to produce an entrained rhythm of clock activity (Greenham and McClung, 2015; Laosuntisuk et al., 2023). Thus, we analyzed the promoter activities of *CCR2* (an evening-phase gene) and *CCA1* (a morning-phase gene) under LD conditions at 14, 21, 28, and 35 DAS. Unlike under circadian conditions (i.e., DD and LL), the diurnal oscillation of *CCR2* and *CCA1* promoter activities exhibited 24-hour periods under LD across all age stages (**Supplementary Figure 2**). This indicated that the circadian clock was reset by the daily light-dark cycle, as previously reported (Takahashi et al., 2015).

**Figure 2:**
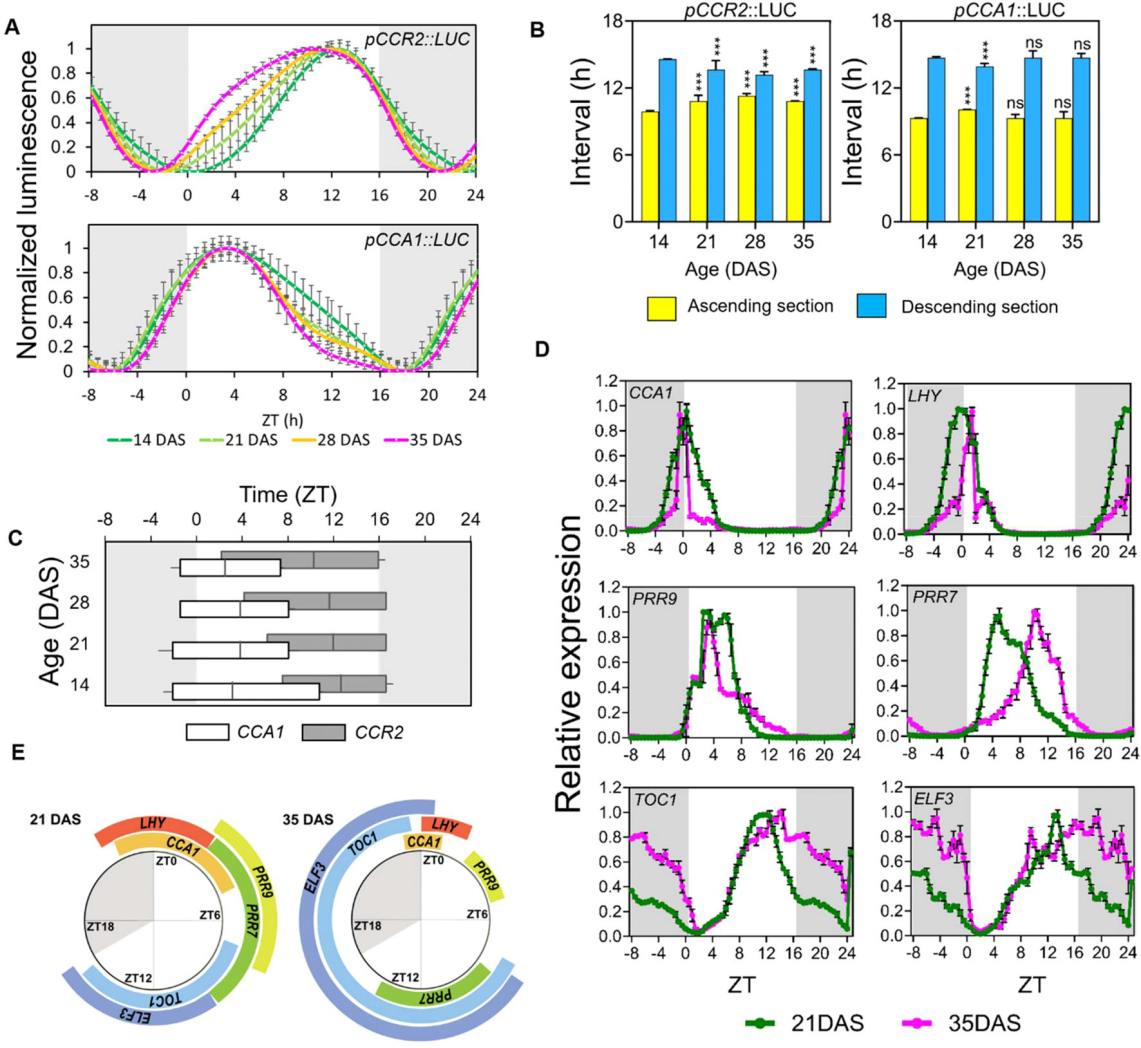
Age-dependent rhythmic alteration of the daily expression schedule of circadian clock components. **(A)** Age-dependent changes of promoter activities of pCCR2::LUC and pCCA1::LUC (relative intensity ± SD), respectively, under long-day conditions. Luminescence signals were normalized for amplitude and detrended across the first three cycles to display the differences in period and waveform (n = 16 leaves for each condition). **(B)** The duration (hours ± SD) of theascending (yellow) and descending (blue) sections; ***p-value<0.001 using two-way ANOVA and Dunnett’s multiple comparison test (n = 16 leaves)against each interval of 14 DAS samples. **(C)** Age-dependent changes in the FWHM values (hours ± SD). The FWHM values of pCCR2::LUC (upper) and pCCA1::LUC (lower) are shown along with the crest time points. **(D)** Daily expression patterns of major clock components under long-day conditions. Theexpression (relative expression ± SD) of the circadian clock components at 21 (green) and 35 (magenta) DAS was measured using qRT-PCR with a time resolution of 30 min. The curves are normalized by the average values of amplitudes across time points to better display differences in period and waveform (n = 3biological replicates for each condition). The expression data from ZT 1 to 8 were duplicated and placed in ZT 25-32 for visualization. **(E)** The dailytimetable of FWHMs of clock components. The FWHMs of each gene in (D) were plotted around a daily schedule to show the changes in durationand timing of FWHM between 21 (left) and 35 (right) DAS plants. **(A–D)** Data are represented as mean ± SD. ns, not significant. See also **Supplementary Figure 2** and **Supplementary Figure 3**.

Despite the identical periods across samples, aged leaves showed broadened curves of diurnal oscillations of *CCR2* promoter activity predominantly in the ascending section, with peaks shifting toward dawn (**Figure 2A**). This shows an age-dependent rhythmic alteration of *CCR2* promoter activity rhythm under LD. Notably, this rhythmic alteration pattern under LD is distinct from that under DD: (1) minimal peak phase shift under LD compared to significant phase shift under DD, and (2) curves broadening under LD but narrowing under DD (**Figures 1A**, **2A**). Binary sectional analysis of diurnal *CCR2* promoter activities showed that the ascending section increased with age, while the descending section decreased (**Figure 2B**), This contrasts with the rhythmic alteration patterns observed under DD where the durations of the ascending and the descending sections decreased and increased, respectively, with age. The FWHM analysis also showed that the descending time-points of the *CCR2* expression occurred consistently at Zeitgeber Time (ZT; the time in hours from lights-on) 16 or 17, regardless of age. However, the ascending time-points advanced with age, from ZT 6.9 at 14 DAS to ZT1.98 at 35 DAS, extending the FWHM length from ZT9.4 h to 14.8 h (**Figure 2C**). This indicates that the daily activation (ascending phase) of *CCR2* expression (and potentially other evening genes) starts earlier under LD as aging progresses (**Figure 2C**), a pattern less pronounced under DD.

Interestingly, the rhythmic alteration patterns of diurnal *CCA1* promoter activities differed from those of *CCR2* (**Figure 2A**). For example, *CCA1* expression showed a narrowing of the curve, mostly in the descending section, and a shift toward morning, while *CCR2* showed a broadening of the curve, mostly in the ascending section, also with a shift toward morning. Although binary sectional analysis of the *CCA1* promoter showed only minor changes in the ascending and descending sections (**Figure 2B**), FWHM analysis revealed minimal changes in the ascending time-points, but substantial changes in descending time-points (ZT11.7 at 14 DAS to ZT7.6 at 35 DAS), resulting in a shortening of FWHM duration from 12.3 h at 14 DAS to 8.5 h at 35 DAS (**Figure 2C**). These findings suggest that the pattern of age-dependent rhythmic alteration in the circadian clock is gene-specific.

Next, we investigated whether age-dependent rhythmic alteration observed in the diurnal promoter activity of *CCA1*, one of the core clock genes, is widespread among core clock components and, if so, how different their rhythmic alteration patterns are. We selected six core clock component genes with different expression peaks (Alabadi et al., 2002, 2001; Box et al., 2015; Nakamichi, 2011; Yang et al., 2020): the evening components *EARLY FLOWERING 3* (*ELF3*) and *TOC1*, and the morning components *CCA1*, *LONG ELONGATED HYPOCOTYL* (*LHY*), *PSEUDO-RESPONSE REGULATOR 7* (*PRR7*), and *PRR9*. Diurnal expression analysis was performed using quantitative real-time polymerase chain reaction (qRT-PCR) analysis on the third leaves of 21 DAS and 35 DAS plants, with samples collected at 30-min intervals (**Supplementary Figure 3A**). After normalizing the expression levels across time-points due to reduced amplitudes at 35 DAS relative to those at 21 DAS (**Supplementary Figure 3B**), our comparative analysis of diurnal waveforms revealed age-dependent rhythmic alteration in gene expression patterns (**Figure 2D**). Consistent with the reporter assay (**Figures 2A, B**), the expression duration of *CCA1* was shorter at 35 DAS compared to the one at 21 DAS. Similarly, other morning-phase genes (*LHY1* and *PRR9*) exhibited decreases in FWHM durations while those of the evening genes, *ELF3* and *TOC1*, showed increased FWHM durations in aged plants (**Supplementary Figure 3C**). We also measure the diurnal expression of *CCR2* in the same samples using qRT-PCR, revealing consistent changes in FWHM duration observed in its promoter activities (**Supplementary Figure 3D**). An exception was *PRR7*, which showed a complete shift in expression timing: the FWHM occurred between ZT 2.5 and 9.5 at 21 DAS, but occurred between ZT 8.5 and 14.0 at 35 DAS (**Figure 2E**). Collectively, these results indicated that the rhythmic alteration of gene expression patterns in aged plants under diurnal conditions occurred in various core circadian component genes, shortening the expression duration of morning-phase genes and lengthening that of evening-phase genes often associated with phase shift along leaf aging.

### 3.3 Age-dependent alterations in diurnal transcriptomes

Based on the finding that various circadian clock core component genes exhibited age-dependent rhythmic alteration of their diurnal expression patterns, and considering that many of these genes are transcription factors (Greenham and McClung, 2015), we hypothesized that age-dependent rhythmic alteration is not limited to core clock component genes, but is a global phenomenon. To test this, we performed RNA-seq on the third leaves of plants at 21 and 35 DAS plants collected at six different time-points over a 24 h period (ZT1, 5, 9, 13, 17, and 21) under LD conditions (**Supplementary Data Sheet 1**). Using our computational pipeline, we identified 19,752 and 19,769 genes expressed across the 24 h period in 21 and 35 DAS plants, respectively. Among these, we identified 8,201 (41.5%) and 2,655 (13.4%) genes as daily cycling genes at 21 and 35 DAS, respectively using the widely used algorithm CosinorPy (Moskon, 2020) for rhythmicity detection and analysis, (see Methods) (**Supplementary Data Sheet 2**). The number of cycling genes at 21 DAS was comparable to previous report (Yang et al., 2020) while the number decreased to 32.3% at 35 DAS (**Supplementary Figure 4A**). By comparing these gene sets, we found that 2,154 genes were expressed at both ages, whereas 6,047 and 501 genes showed oscillations only at 21 or 35 DAS, respectively. This indicated that transcriptomic responses to the light-dark cycles attenuated as aging progressed, consistent with the reduced amplitude of diurnal expression rhythms observed in the six clock genes analyzed earlier (**Supplementary Figure 3B**). These findings indicate that physiological processes in aged plants are under less stringent oscillatory regulation than in young plants. Similarly, the numbers of commonly oscillating genes with similar, higher, or lower amplitudes at 35 DAS compared to 21 DAS were 662, 61, and 237, respectively, indicating that a large proportion of the commonly oscillating genes underwent a reduction in expression (**Supplementary Figure 4F**). The daily expression patterns of cycling genes included those with more than one peak within a 24 h period. Thus, to accurately compare the rhythmic alteration patterns of cycling genes in young and old plants, we used the permutation test (Jean-Richard-Dit-Bressel et al., 2020) to identify genes that produced only a single FWHM, referred to as “single oscillation genes (SOGs)”, revealing 4,134 and 1,458 SOGs expressed at 21 and 35 DAS, respectively (**Supplementary Figures 4B, C**). Comparison of SOGs between 21 and 35 DAS plants showed that 960 SOGs overlapped (common SOGs) across both ages, whereas 3,174 and 498 genes showed a single oscillation preferentially at 21 DAS and 35 DAS, respectively (**Supplementary Data Sheets 3** and **5**).

Gene ontology (GO) analysis (Huang et al., 2009) of the commonly oscillating genes identified “circadian rhythm” as the top-scoring term (**Supplementary Figure S4D**), indicating the significance of the circadian clock in controlling the daily expression cycle at both ages. Environmental cues-related pathways were strongly enriched in the oscillating genes expressed preferentially in young plants, while no GO terms were enriched among the oscillating genes preferentially expressed in old plants, indicating that these genes did not act in specific pathways. Additionally, GO analysis of SOGs (**Supplementary Figure S4E**) revealed “response to light stimulus” as the top-scoring term in both common and young preferential SOGs while metabolic and wounding responses were moderately yet significantly enriched in old preferential SOGs. These findings indicate not only the key roles of circadian and light-related functions but also the significant involvement of other biological pathways in the diurnal transcriptomic oscillations.

### 3.4 Age-dependent changes in diurnal expression patterns of senescence-regulatory genes in plant leaves

A critical physiological outcome of aging in plant leaves is senescence, which is followed by cell death. Regulation of age-dependent leaf senescence involves changes in the expression of senescence regulatory genes that control downstream genes in the senescence signaling transduction process (Kim et al., 2018b). We examined the diurnal expression patterns of known senescence-related genes in our transcriptomic dataset (**Supplementary Figure 5A**, **Supplementary Data Sheet 4**).

From the leaf senescence database (Li et al., 2020), we identified 353 senescence regulator genes, of which 170 showed daily oscillations in expression. Among these, 71 negative and 61 positive regulator genes were preferentially expressed in young plants, while only two negative and three positive regulator genes were preferentially expressed in old plants. We compared the FWHMs of the 33 SOGs encoding senescence regulators in both young and old plants (**Supplementary Figures 5B, C**). Among these, three negative and four positive regulators showed significant FWHM shortening in the diurnal expression at 35 DAS compared to 21 DAS, while two negative and two positive regulator genes showed significant lengthening of FWHM. For example, *MYC2*, encoding a positive regulator involved in JA-mediated defense and leaf senescence, exhibited a significant rhythmic alteration of its oscillatory pattern with an extended FWHM at 35 DAS during the day (**Supplementary Figure 5**). These results revealed that 33% (11 out of 33) SOGs encoding senescence regulators exhibit age-dependent changes in their diurnal expression oscillation.

### 3.5 Age-dependent rhythmic alteration of daily gene expression rhythms occurs at the transcriptomic level

**Figure 3:**
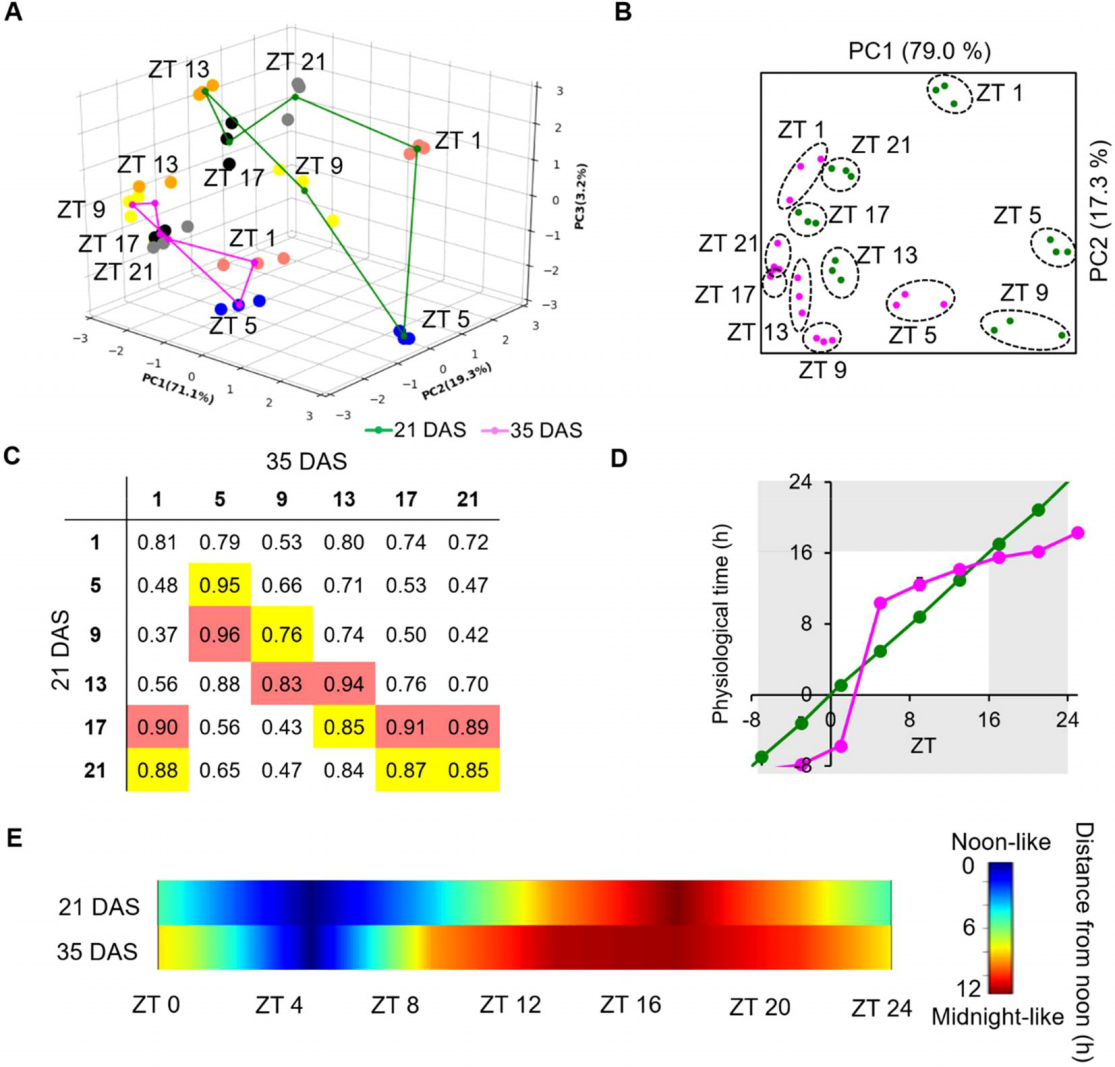
Transcriptome-level rhythmic alteration of the diurnal expression schedule of genes with a single common oscillation in old plants. **(A)** Threedimensional PCA of the transcriptomic data from 21 DAS (green lines) and 35 DAS (magenta lines) plants. The transcriptomic data were obtained from the 3rd leaves of 21 and 35 DAS plants collected at 4 h intervals across 24 h; the (n = 3). Dots represent the transcriptomic characteristics of the samples and are colored according to the sampling time: red: ZT1; blue: ZT5; yellow: ZT9; orange: ZT13; black: ZT17; and gray: ZT21. **(B)** Twodimensional PCA analysis of the transcriptomes following normalization of the amplitude. **(C)** Correlation matrix of the transcriptome characteristicsof 21 and 35 DAS samples at each time-point. The best and second-best matches between the two transcriptomes are highlighted in red andyellow, respectively. **(D)** Change of subjective time of the 35 DAS transcriptome relative to the 21 DAS transcriptome. **(E)** Diagram depicting the change in subjective time across 24 (h) See also **Supplementary Figures 6** and **7**.

To further investigate age-dependent rhythmic alteration in the expression patterns of the 960 common SOGs (**Supplementary Figure 4B**), we analyzed the landscape of diurnal transcriptomic rhythms, which are characterized by amplitude, phase, and waveform (Castells et al., 2007). Note that the period is reset to 24 h every day under the diurnal cycle of our experimental condition (**Figure 2**). We first performed a three-dimensional principal component analysis (PCA) on the expression profiles of these genes to capture the diurnal characteristics (**Figure 3A**). The PCA revealed high reproducibility, with biological replicate samples clustering tightly at each time-point for a given age. While samples were separated by time-points, indicating dynamic transitions over the 24 h LD cycle, the age factor predominantly clustered samples (i.e., 21 DAS vs. 35 DAS). Rotations of the 3D projection further confirmed clear differentiation between young and old plants in the cycling planes of the projected transcriptomes (**Supplementary Figure 6A, B**), likely due to the attenuated amplitudes in the aged leaves as shown by the clock gene expression (**Supplementary Figure 5C**). To make the two sample sets comparable, we normalized the expression values of each gene across time-points using the average values at each aging stage, merging the two separated cycling planes (**Supplementary Figure 6C**). Following normalization, two-dimensional PCA revealed that diurnal samples primarily clustered based on the time of day (**Figure 3B**). Notably, young and old plants exhibited differences. For example, ZT9, ZT13, ZT17, and ZT21 samples clustered more tightly in 35 DAS plants than in 21 DAS plants, indicating attenuation of the 960 common SOGs expression across these time-points as plants aged. Additionally, the influence of time-points on the diurnal transcriptome was altered: ZT 1 samples at 35 DAS were closer to ZT21 and ZT17 samples at 21 DAS than to ZT1 samples, while ZT9 samples at 35 DAS were closer to ZT13 at 21 DAS samples than to ZT9 samples (**Figure 3B**). This was consistent with Pearson’s correlation coefficient (PCC) analysis, which showed the highest correlation of ZT1 samples at 35 DAS with ZT17 (PCC = 0.90) or ZT21 (PCC = 0.88) at 21 DAS, and the highest correlation of ZT9 at 35 DAS with ZT13 (PCC = 0.83) at 21 DAS. These results indicated that the physiological states plants encounter across the 24 h LD cycle was affected as plants aged, potentially leading to rhythmic alteration of the gene expression rhythm.

Using ridge regression analysis (Marquardt and Snee, 1975)previously employed to estimate “physiological time” in *Drosophila melanogaster* (Litovchenko et al., 2021), we estimated the physiological time of the transcriptomic profiles at each time-point by comparing samples between 21 DAS and 35 DAS, using the 21 DAS samples as the reference (**Supplementary Figure 7**; **Figure 3D**). At 35 DAS, the daily expression transitions of the 960 common SOGs exhibited a unique pattern: a steep surge between ZT1 and ZT5, corresponding to 16.1 h span at 21 DAS, followed by a slow transition over the 16 hours between ZT5 and ZT21 that corresponded with only a 7.9 h transition at 21 DAS (**Figure 3D**). We then calculated the distance of the 35 DAS transcriptome from the “noon” transcriptome at 21 DAS at each ZT (**Figure 3E**), revealing that daytime was shorter and nighttime longer at 35 DAS compared to 21 DAS (**Figure 3E**). Our data suggest that age-dependent rhythmic alteration occurs at the transcriptome level, resulting in aged plants experiencing a shorter day and longer night.

### 3.6 Genes with daytime and nighttime peaks undergo rhythmic alteration in opposite directions in aged plants

**Figure 4:**
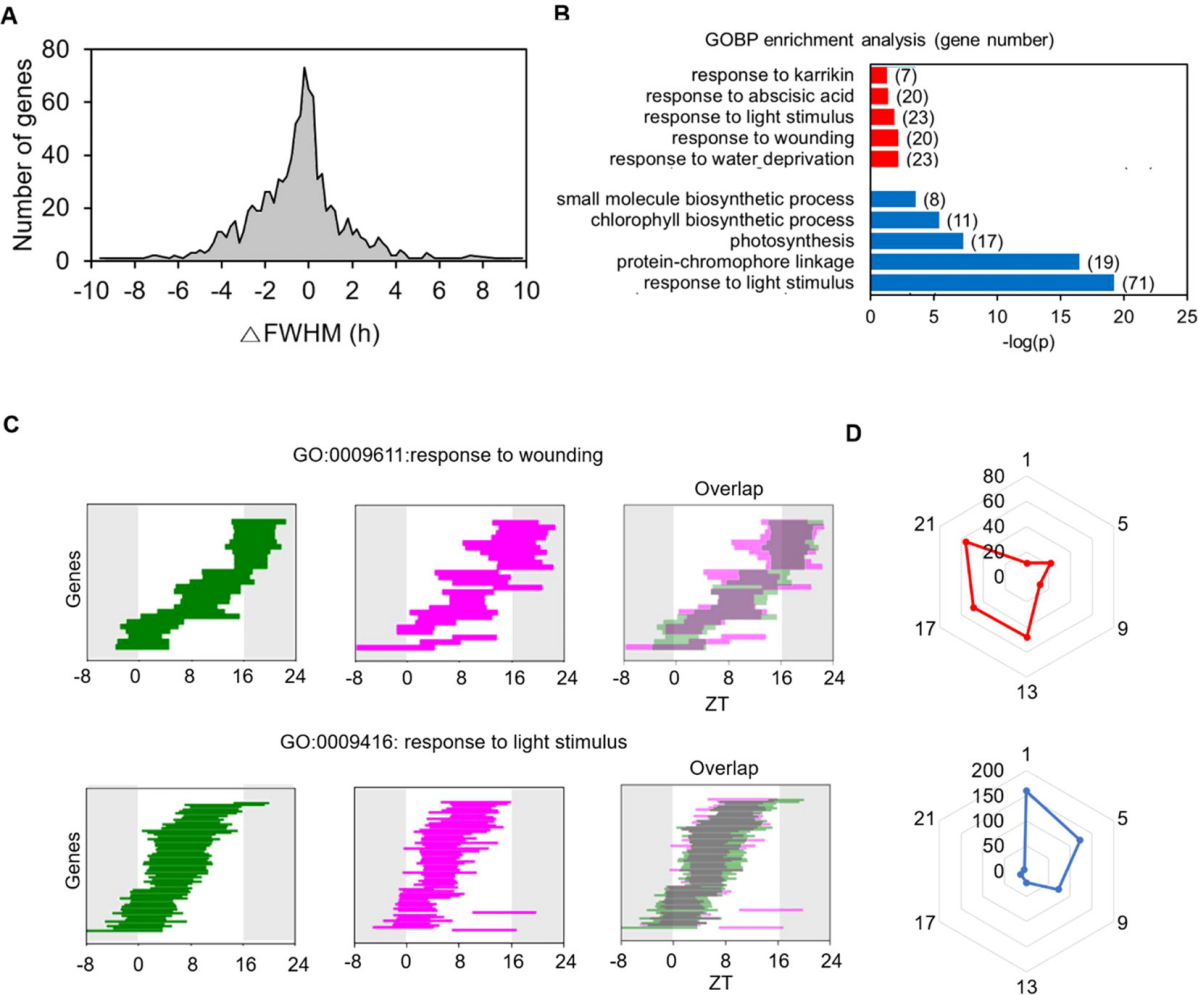
Age-dependent changes in FWHM of genes with common single daily oscillations at 21 and 35 DAS. **(A)** Histogram of changes in FWHMs in 35 DASplants compared to those in 21 DAS plants. The FWHM of each gene is the mean value of the cycling curves generated by the permutation of replicates for each time point. **(B)** Gene Ontology (GO) categories enriched among genes with changed FWHM at 35 DAS. The GO analysis of genes with increased (red) and decreased (blue) FWHM values is shown. **(C)** Changes in FWHM values of genes involved in the GO categories “response to light stimulus” and “response to wounding”. The FWHM analyses at 21 (green) and 35 (magenta) DAS and their overlap patterns are shown. **(D)** Polar plots for the number of genes with increased (red) and decreased (blue) FWHM values for genes showing maximum expression at a given time point. See also **Supplementary Figures 4** and **5**.

To better understand the age-dependent rhythmic alteration occurring at the transcriptome level and its biological function, we conducted an FWHM analysis on the 960 common SOGs. Among these, 282 (29.4%) exhibited a significant change in FWHM length at 35 DAS compared to 21 DAS (*P* < 0.05 and |ΔFWHM| > 2 h in the permutation test). Of these 282 genes, 194 showed a decrease in FWHM length, and 88 showed an increase, indicating a shortened and lengthened period of expression period at 35 DAS during the LD cycle over 24 h (**Figure 4A**). This data indicates that the daily expression times of the common SOGs altered with age. GO analysis revealed that the “responses to light” category was enriched among genes with shortened daily expression periods. This suggests that, despite the light/dark regime remaining constant, older plants responded to light for a shorter duration than younger plants. Conversely, enriched categories among genes whose daily expression time lengthened were primarily related to stress responses, including “response to water deprivation”, “response to wounding”, and “response to abscisic acid” (**Figure 4B**). The FWHM analysis of genes in the “response to light stimulus” and “response to wounding” categories illustrated how the daily expression schedule of these gene groups changed (**Figure 4C**). Genes in the “response to light stimulus” category were mainly expressed during the daytime, but their FWHM range was substantially reduced at 35 DAS. In contrast, The FWHMs of genes in the “response to wounding” category spanned the entire 24 h period, and their duration of expression expanded at 35 DAS.

We created two polar charts of peak time for genes with lengthened and shortened FWHMs to examine the overall rhythmic alteration patterns of the 960 common SOGs (**Figure 4D**). Genes with decreased FWHM values in older plants were enriched at ZT1, while genes with increased FWHM values were enriched at ZT21. These findings align with the changes in FWHM values for the core clock genes (**Figure 2E**). Genes with expression peaks during the day (ZT1) and at night (ZT21) exhibited opposite rhythmic alteration directions as the plants aged: the expression durations of common SOGs peaking during the day shortened, whereas those peaking at night extended. This suggests that the rhythmic alteration of diurnal gene expression patterns upon aging depends on their daily expression time and represents a general mode of regulation among the common SOGs.

### 3.7 Rhythmic alteration of daily gene expression rhythms is mediated through selected core clock components

**Figure 5:**
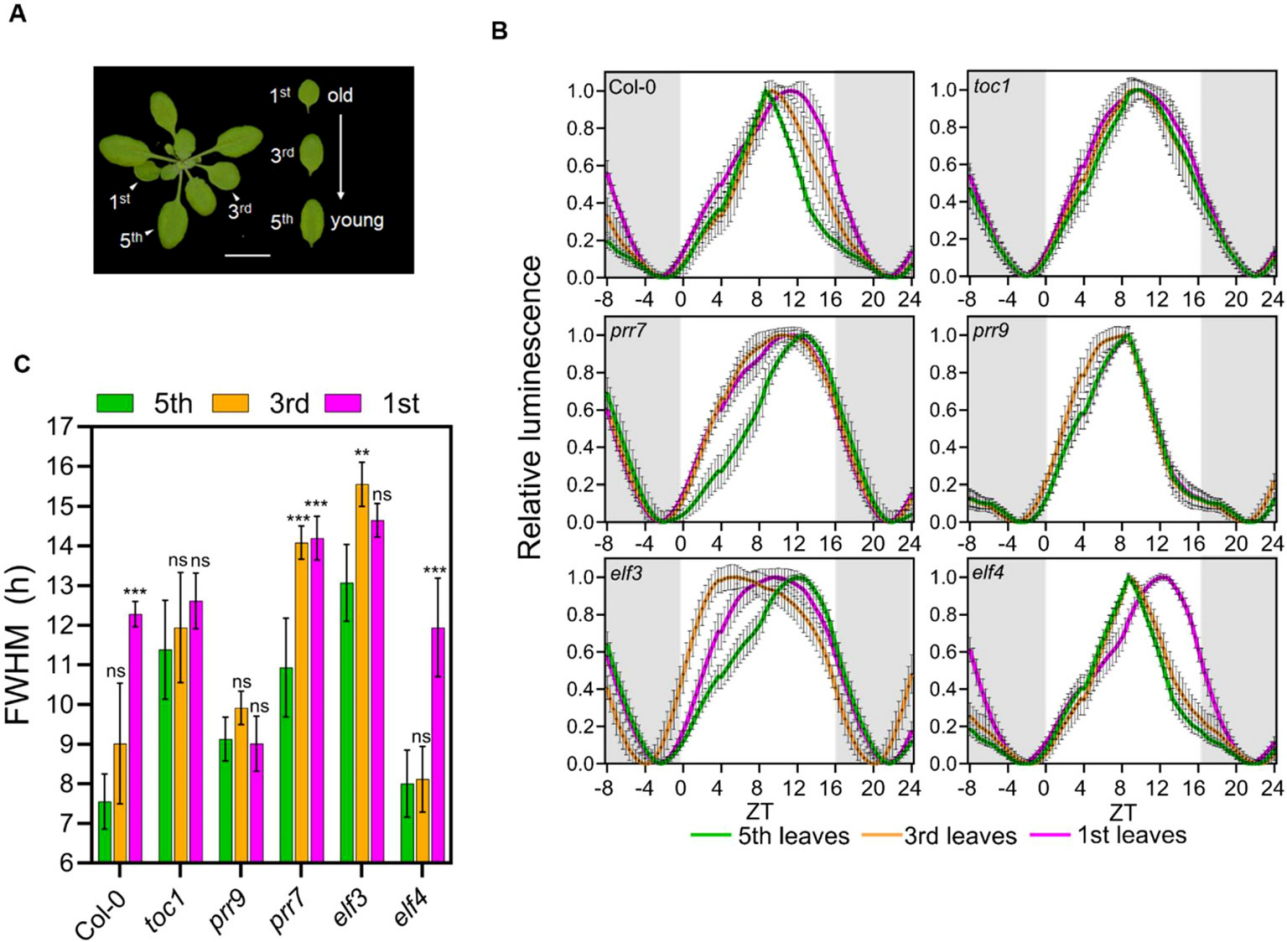
Genetic effects on age-dependent rhythmic alteration of daily gene expression patterns. **(A)** Leaves with different developmental stages used in the assay. Scale bar, 1 cm. **(B)** Daily rhythms (relative intensity ± SD) of pCCR2:LUC in circadian clock mutants under long-day conditions. Luciferase activities in plantsharboring pCCR2:LUC are shown; expression was normalized on amplitude between ZT24 and ZT52. These leaves had different chronological agesdue to differences in their emergence time. The initial expression data from ZT1 to ZT12 were duplicated and placed in ZT25-36 for visualization. **(C)** Age-dependent changes in FWHM values (hours ± SD) in circadian clock mutants; **p-value < 0.01,*** < 0.001 using two-way ANOVA and Dunnett’s multiplecomparison test (n = 16 leaves). **(B, C)** Data are represented as mean ± SD. See also **Supplementary Figure 8**.

So far, we have found that the duration of daytime-associated gene expression is shorter in older plants compared to younger plants under LD (**Figure 4**) and that the expression of core clock genes exhibited rhythmic alteration (**Figure 2**). These findings led us to hypothesize that core clock genes regulate the rhythmic alteration of diurnal rhythmic expression. To test this hypothesis, we investigated the activity of *pCCR2::LUC* reporter in the 1^st^, 3^rd^, and 5^th^ leaves of a few clock mutants, *toc1*, *prr7*, *prr9*, *elf3*, and *elf4* at 21 DAS and compared them to the wild-type Col-0 (**Figures 5A**; **Supplementary Figure 8**). By measuring the clock activities in a series of leaves at identical developmental stages across various genotypes, we were able to eliminate the potential effects of the genetic mutations on plant aging. In Col-0, the diurnal curve of *pCCR2::LUC* was broadened towards dusk as the leaves aged, which is validated by an increased FWHM (**Figure 5B, C**). In contrast, two circadian mutants *toc1* and *prr9* showed no significant change in *pCCR2::LUC* diurnal activity with age, indicating that TOC1 and PRR9 play key roles in generating rhythmic alteration. The daytime activity of *pCCR2::LUC* extended towards dawn in *elf3* and *prr7* mutants in contrast to the dust-oriented extension in wild-type plants, suggesting a function of these genes in directing rhythmic alteration patterns. The *pCCR2::LUC* diurnal activity was least affected in the *elf4* mutant. Furthermore, since flowering is one of pathways associated with the circadian clock and aging (Johansson and Staiger, 2015), we conducted FWHM analysis of CO and FT in young (21 DAS) and old (35 DAS) leaves (**Supplementary Figure S9**). Interestingly, both CO and FT showed no significant differences in their FWHM values between the two age groups. This suggests that while flowering is an aging marker, its regulation by the clock may be more related to the emergence of new organs (i.e., flowers) rather than preparation for senescence. Therefore, flowering seems to follow a pathway independent of the clock-associated aging mechanism demonstrated in this study. Collectively, our genetic analysis demonstrates that core clock components broadly affect changes in daily oscillations seen in aged plants. This suggests that the alterations in rhythmic gene expression in aged plants result from the combined effects of the external environmental cycle and the endogenous circadian rhythm.

## 4 Discussion

Interplays between the circadian clock and plant senescence machinery has been have been reported, including by our group, as gene expression patterns alter significantly in aged plants, yet has not been characterized despite the biological significance. In this study, we revealed the existence of the age-dependent rhythmic alteration in Arabidopsis under both circadian and diurnal conditions, termed as rhythmic alteration. We further characterized age-dependent changes in rhythmic patterns of gene expression under diurnal conditions on a global scale, demonstrating that diurnal rhythms of clock marker gene expression undergo rhythmic alteration in a phase-specific manner, distinct from the patterns observed in circadian conditions. Our transcriptomic analysis revealed that this age-dependent rhythmic alteration resulting in aged plants experiencing a shorter day and longer night. Our genetic analysis further demonstrated that various circadian clock components regulate this age-dependent rhythmic alteration of rhythms. Thus, our integrative approach combining reporter assay, transcriptome analysis, and genetics revealed that diurnal gene expression rhythms controlled by core clock components globally differ greatly between young and aged plants and these age-dependently altered clock activities may play an important role in plant senescence.

The followings are the characteristic changes of diurnal rhythmic gene expression in aged Arabidopsis plants we observed in our study and their implication in senescence physiology. First, our transcriptome analysis revealed that the number of diurnally oscillating genes is far reduced in aged plants. In our experimental condition, the number of oscillating genes was only 2,655 (13.4%) in old plants compared to 8,201 (41.5%) in young plants despite of similar number of genes expressed at both ages. The observation indicate that the reduced oscillation of overall transcriptome characterizes a part of senescence physiology in plant leaves, implying that fewer physiological processes are regulated and coordinated by the daily rhythm in aged plants. We argue that at least in part this is due to the off-resonance between the internal circadian rhythm and the environmental light rhythm. Second, different GO terms were enriched in cycling genes between older and younger plants, indicating changes in rhythmic physiology. Furthermore, there are sets of genes oscillating specifically at the young or old stages. Among the 8,201 and 2,655 genes oscillating at young and old plants, respectively, 6,047 and 501 genes showed oscillations specifically in young and old plants, respectively. The result indicated that these differences in gene sets of oscillating in part characterize the senescence physiology in aged plants. Third, our focus in this report was “rhythmic alteration” or the change of the waveforms of the rhythmic patterns of daily cycling genes in aged plants compared to those in young plants. Previously, the characterization of the oscillating genes was based on their amplitudes, periods, and phases. However, here we found that rhythmic alteration, which a collective term reflecting changes on the rhythmic parameters in this study and others (van Bree et al., 2022; Williams et al., 2020), is another factor that should be considered in the analysis of rhythmic gene expression in aging plants.

The functional activity of a gene depends not only on its expression level but also on the timing and duration of its expression. Rhythmic alteration of gene expression rhythms alters the functional timing of genes. For instance, MYC2, a positive senescence regulator and a key factor in the upstream cascade of responses to the plant hormone JA (Zhang et al., 2018), showed an increase in FWHM from 5.2 h at 21 DAS to 12.6 h at 35 DAS (**Supplementary Figures 5B, C**). This indicates that the duration of the response to JA increased with age and extends further into the daytime. As MYC2 regulates JA-mediated defense and leaf senescence responses, these rhythmic alteration patterns of rhythmic expression and altered FWHM could adjust defense and leaf senescence strategies in aged plants. We initially observed rhythmic alteration of a circadian reporter gene, *CCR2*, under a free-running circadian condition and a light-driven diurnal condition. The rhythmic alteration patterns in these two conditions were far different. The light cycle oppositely shifted the rhythmic alteration for the *CCR2* expression. Examination of the rhythmic alteration patterns of a few core circadian clock genes revealed that the rhythmic alteration directions (i.e., toward dawn or dusk) in aged plants depend on the daily phase of the genes. Our transcriptomic analysis also revealed that this phase-dependent rhythmic alteration renders aged plants to experience a shorter day and longer night. Furthermore, we demonstrated that rhythmic alteration of the rhythmic gene expression is regulated by various circadian clock components, demonstrating that it is a genetically programmed aging event. Thus, rhythmic alteration of daily gene expression rhythms acts as a regulatory layer to adjust the timing of physiological processes in aged plants, particularly by altering the duration of gene expression during the daytime and nighttime. We propose that the Integrative change in cycling diurnal transcriptome patterns, such as amplitudes, periods, phases, and rhythmic alteration, collectively produces the physiological changes associated with aging. We term this observation an “oscillatory code of senescence” in plants.

Our findings have other implications for understanding the link between the circadian clock and plant senescence. First, gene expression levels should be compared at multiple time points across 24 h, as comparing them at only a single time point could be misleading due to age-dependent rhythmic alteration of rhythmic gene expression schedules. Second, the age-dependent rhythmic alteration patterns in rhythmic gene expression under diurnal conditions differ from those under circadian conditions. Since diurnal conditions better reflect natural physiological transitions, incorporating age-dependent rhythmic alteration into the circadian model will improve our understanding of the daily and age-dependent physiological transitions in natural settings. This will help identify the genetic and environmental effects on these transitions, leading to improved plant productivity and stress response. Third, age-dependent rhythmic alteration in diurnal rhythmic gene expression has a limited effect on senescence regulator genes. Although age-dependent leaf senescence is regulated by a complex array of senescence regulators, our analysis showed that only a small subset of these genes exhibited more pronounced oscillation in aged plants. Further investigation is needed to understand how age-dependent rhythmic alteration in the diurnal expression of these senescence regulator genes contributes to senescence, while also considering the possibility of clock- or rhythmic rhythmic alteration-independent mechanisms.

## Data Availability

The datasets presented in this study can be found in online repositories. The names of the repository/repositories and accession number(s) can be found in the article/**Supplementary Material**.
