# Supplementary material for: The genetically programmed rhythmic alteration of diurnal gene expression in the aged Arabidopsis leaves
Source: Front Plant Sci. 2024 Nov 4;15:1481682. doi: 10.3389/fpls.2024.1481682 (PMC11570267; doi:10.3389/fpls.2024.1481682)
Supplement: Supplementary file 1 [file DataSheet1.pdf]

Supplementary Material

Supplementary Data

Supplementary Data 1. Processed RNA-seq reads (CPM normalized by TMM). Daily oscillation RNA-seq read count in 21 and 35 DAS Col-0 in 4h intervals over 24h.

Supplementary Data 2. List of oscillating genes. Genes exhibiting a daily cycling in their expression at 24-h period using CosinorPy, a rhythm analysis tool.

Supplementary Data 3. List of single oscillating genes. Single oscillation Genes have only one ascending and descending. A permutation test was conducted to obtain FWHM for the comparison of the rhythmic alteration patterns between young and old plants.

Supplementary Data 4. List of senescence regulators. Genes involved in regulating senescence that displayed daily oscillations within a 24-hour cycle.

Supplementary Data 5. FWHM of single oscillating genes. Single oscillation genes FWHM, ascending and descending time in 21 and 35 DAS.

Supplementary Data 6. List of primers used in qRT-PCR. Core clock components and Act2 primers for qRT-PCR.

Supplementary Figures

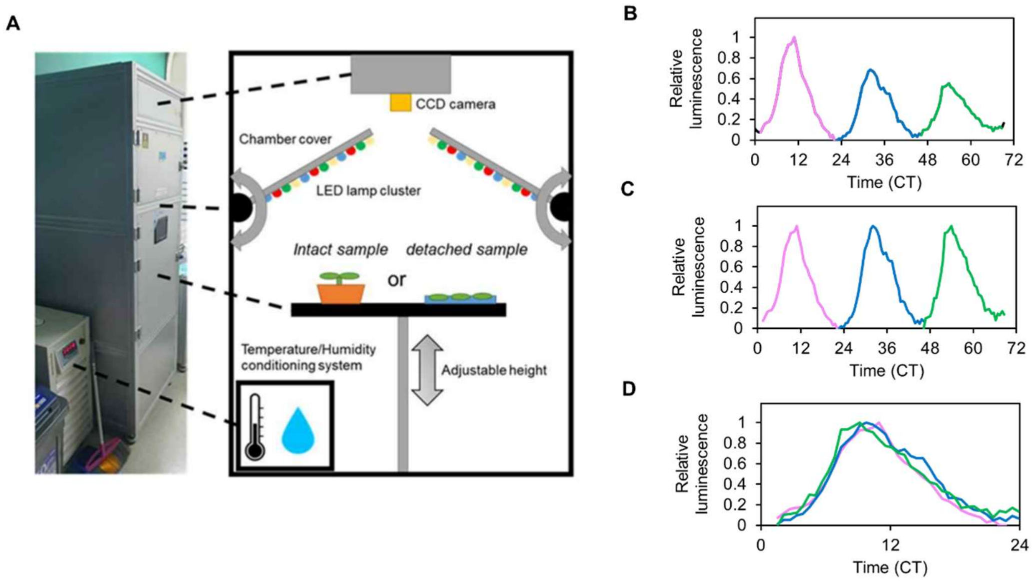

Supplementary Figure 1. Measurement of luminescence imaging and the process of imaging data for monitoring circadian rhythm. **(A)** Luminescence imaging system with a high-resolution CCD camera, humidity sensor, temperature sensor, and LED cluster. **(B)** Mean total luminescence intensity from leaves of *pCCR2::LUC* plants measured in DD after entrainment to LD cycles (16 h light/8 h dark) at the indicated ages. Representative examples at 35 DAS were plotted here. **(C)** Normalization of the circadian curves. Each daily part of the curves was divided by the trough value and was normalized for amplitude**.** **(D)** A detrended and normalized waveform. The circadian rhythms obtained across the first three cycles were merged to generate a detrended waveform.

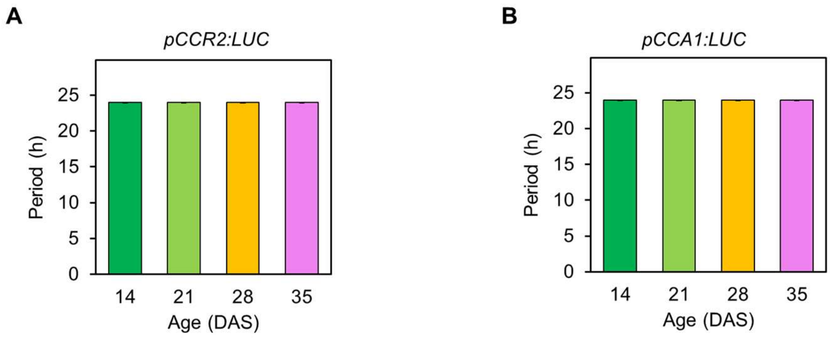

Supplementary Figure 2. The daily cycling periods of *CCR2* and *CCA1* promoter activities under light/dark cycles. **(A,B)** The daily cycling periods of luminescence rhythms of *pCCR2:LUC* (A) and *pCCA1:LUC* (B) expression were measured at different ages under LD conditions.

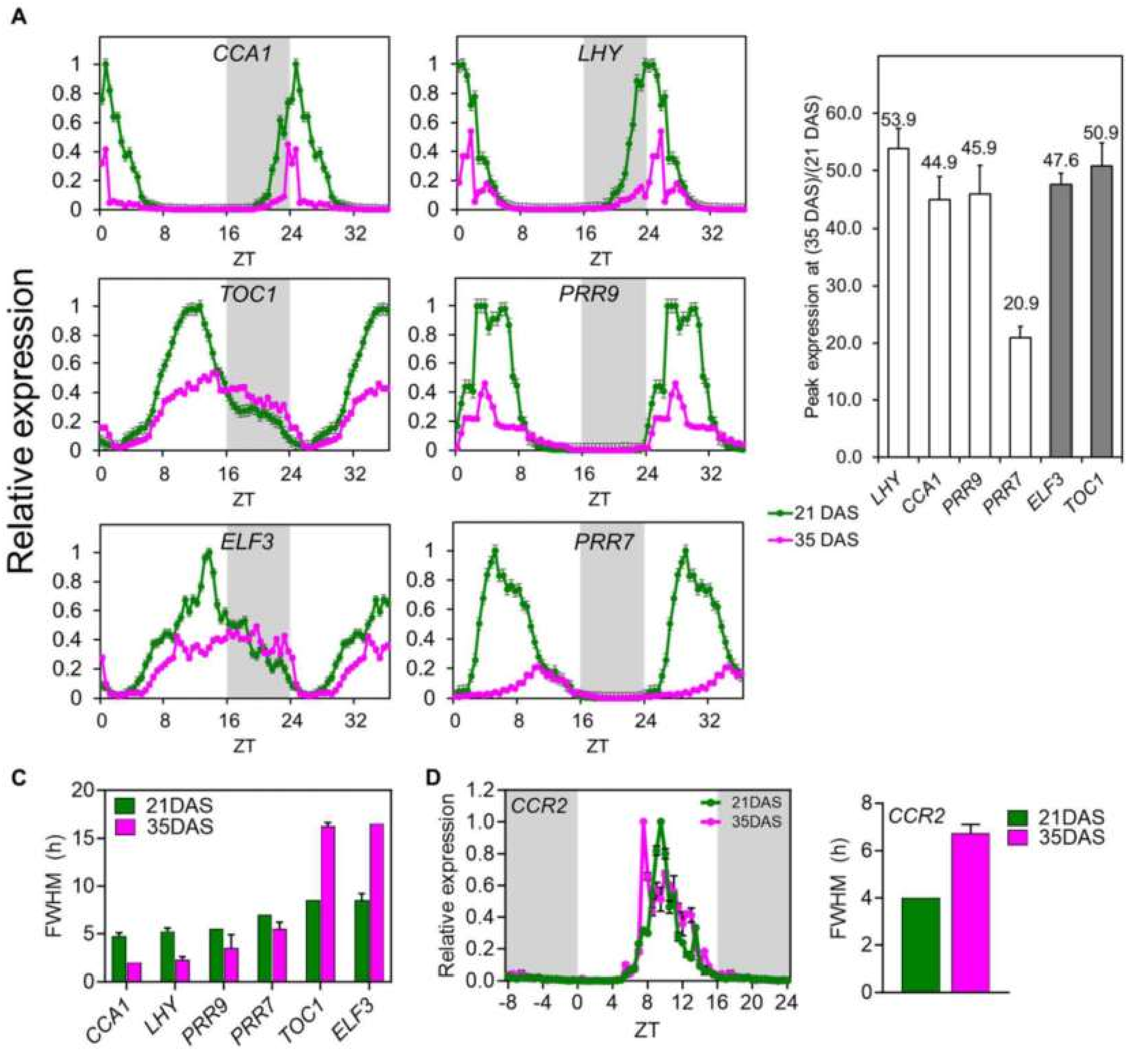

Supplementary Figure 3. Age-dependent changes in the expression patterns of core circadian components. Data are represented as mean ± SD. **(A)** Daily expression patterns of core clock components measured by qRT-PCR with a time resolution of 30 min in 21 and 35 DAS plants grown under LD conditions (16 h light /8 h dark). The second cycle of expression data was duplicated with the first cycle for visualization purposes. **(B)** Peak expression levels of core clock components in 35 DAS plants relative to those in 21 DAS plants; values are normalized on expression in 21 DAS plants. **(C)** The FWHM of core clock components measured by qRT-PCR in 21 and 35 DAS plants. **(D)** Daily expression pattern of *CCR2* measured by qRT-PCR with a time resolution of 30 min in 21 and 35 DAS plants. The expression data was normalized for amplitude (left), and the FWHMs were driven from the normalized daily rhythms (right).

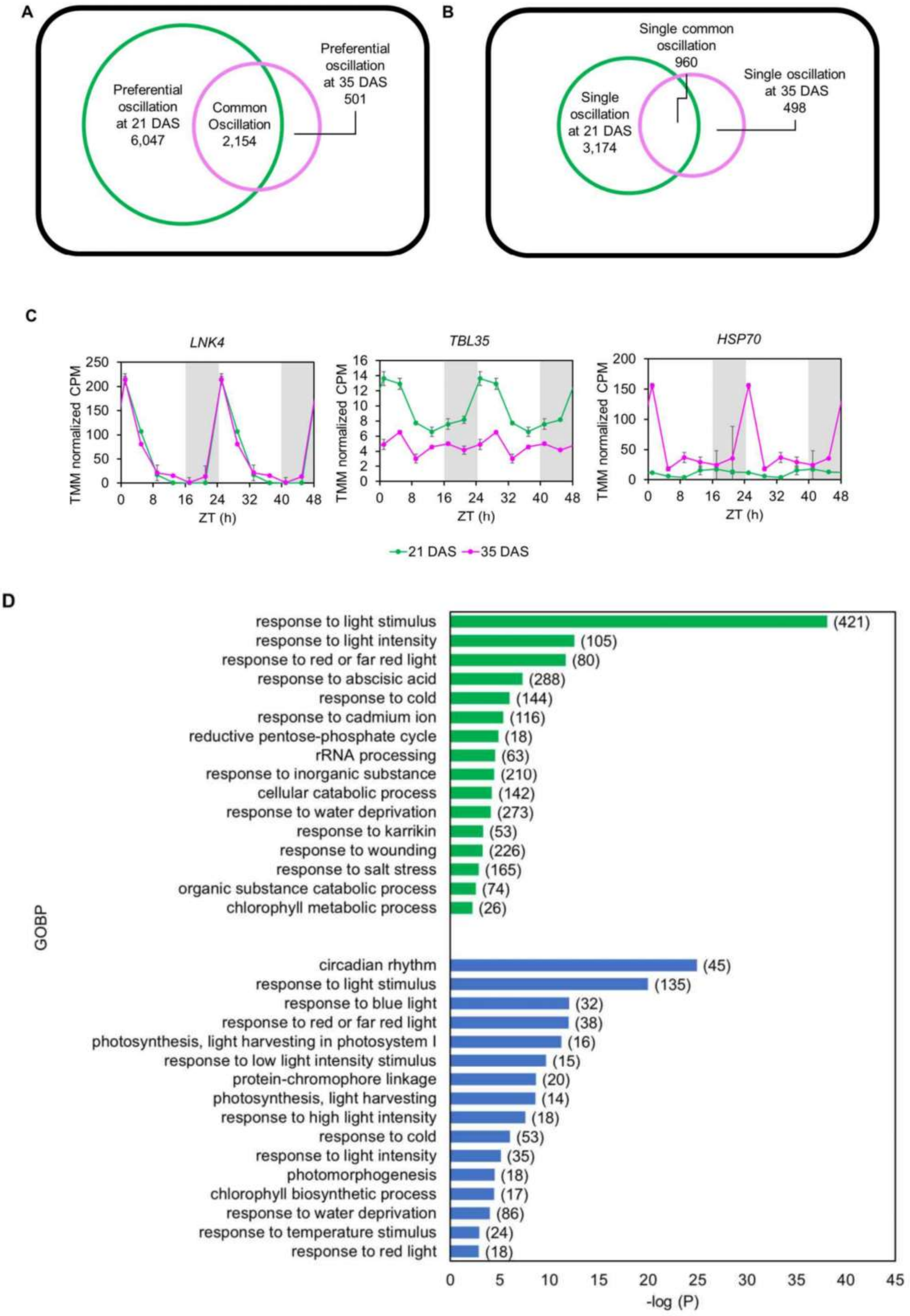
A
Preferential oscillation at 21 DAS 6,047
Common Oscillation 2,154
Preferential oscillation at 35 DAS 501
B
Single common oscillation 960
Single oscillation at 21 DAS 3,174
Single oscillation at 35 DAS 498
C
LNK4
TBL35
HSP70
TMM normalized CPM
ZT (h)
21 DAS
35 DAS
D
GOBP
response to light stimulus (421)
response to light intensity (105)
response to red or far red light (80)
response to abscisic acid (288)
response to cold (144)
response to cadmium ion (116)
reductive pentose-phosphate cycle (18)
rRNA processing (63)
response to inorganic substance (210)
cellular catabolic process (142)
response to water deprivation (273)
response to karrikin (53)
response to wounding (226)
response to salt stress (165)
organic substance catabolic process (74)
chlorophyll metabolic process (26)
circadian rhythm (45)
response to light stimulus (135)
response to blue light (32)
response to red or far red light (38)
photosynthesis, light harvesting in photosystem I (16)
response to low light intensity stimulus (15)
protein-chromophore linkage (20)
photosynthesis, light harvesting (14)
response to high light intensity (18)
response to cold (53)
response to light intensity (35)
photomorphogenesis (18)
chlorophyll biosynthetic process (17)
response to water deprivation (86)
response to temperature stimulus (24)
response to red light (18)
-log (P)

E

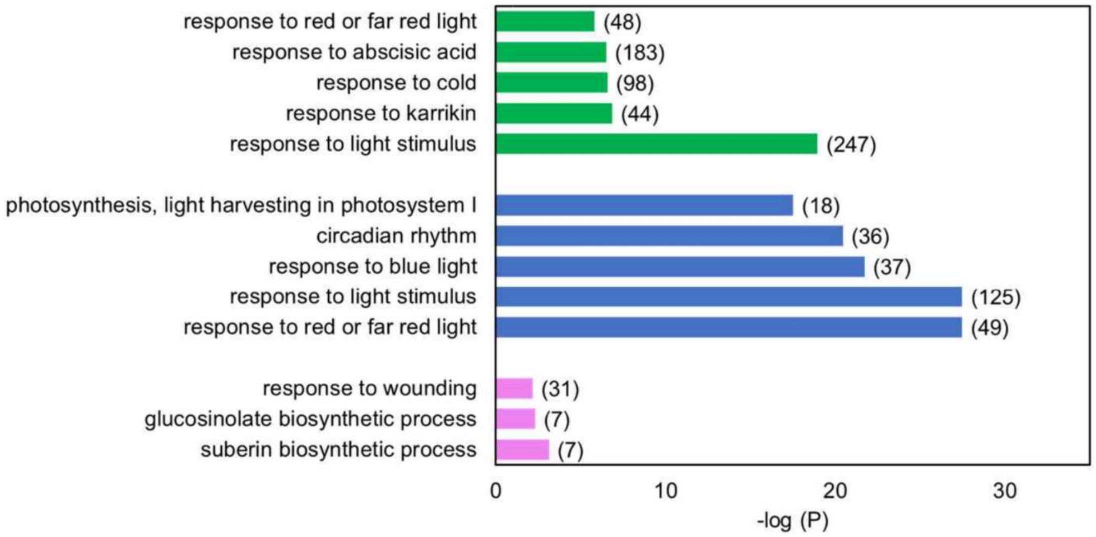

F

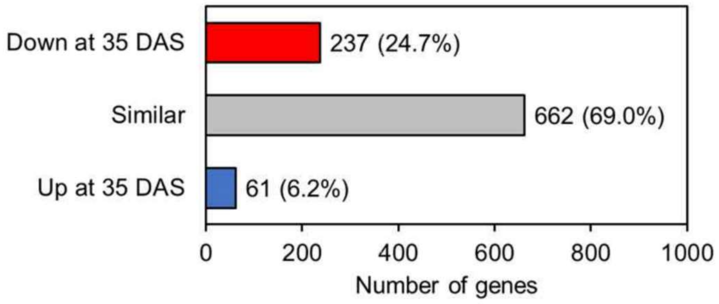

Supplementary Figure 4. Age-dependent changes in oscillating genes in 21 and 35 DAS plants. **(A)** Venn diagram showing the number of genes with oscillations at both ages (common oscillation) and genes showing preferential oscillations at 21 or 35 DAS. **(B)** Venn diagram showing the number of genes showing a single oscillation in a 24 h period at both ages (single common oscillation) and genes showing a single preferential oscillation at 21 or 35 DAS. **(C)** Examples of single oscillation patterns of genes with common (left), young preferential (middle), and old preferential (right) oscillations, *LNK4*, *TBL35*, and *HSP70,* respectively. The initial cycle's expression data was replicated alongside the second cycle. Data are represented as mean ± SD. **(D)** Gene Ontology Biological Process (GOBP) analysis of genes with common (blue), young preferential (green). **(E)** GOBP analysis of single oscillation genes with common (blue), young preferential (green), and old (magenta) preferential oscillations. **(F)** The numbers of commonly oscillating genes with similar, higher, or lower amplitudes at 35 DAS than at 21 DAS (p-value< 0.05 and fold change > 2 or < 0.5).

A

		Numbers of genes
Negative regulators	No oscillation	84
	Young preferential oscillation	71
	Old-preferential oscillation	2
	Common oscillation	14
Positive regulators	No oscillation	99
	Young preferential oscillation	61
	Old-preferential oscillation	3
	Common oscillation	19

B

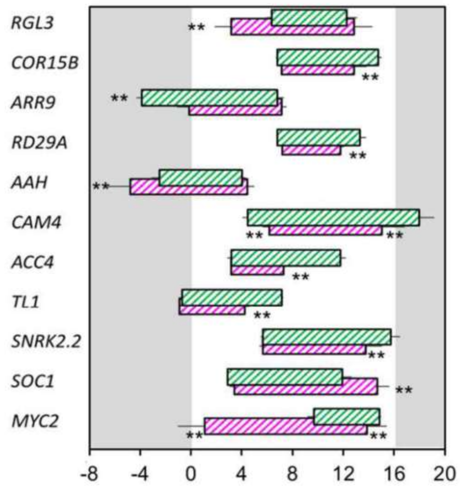

C

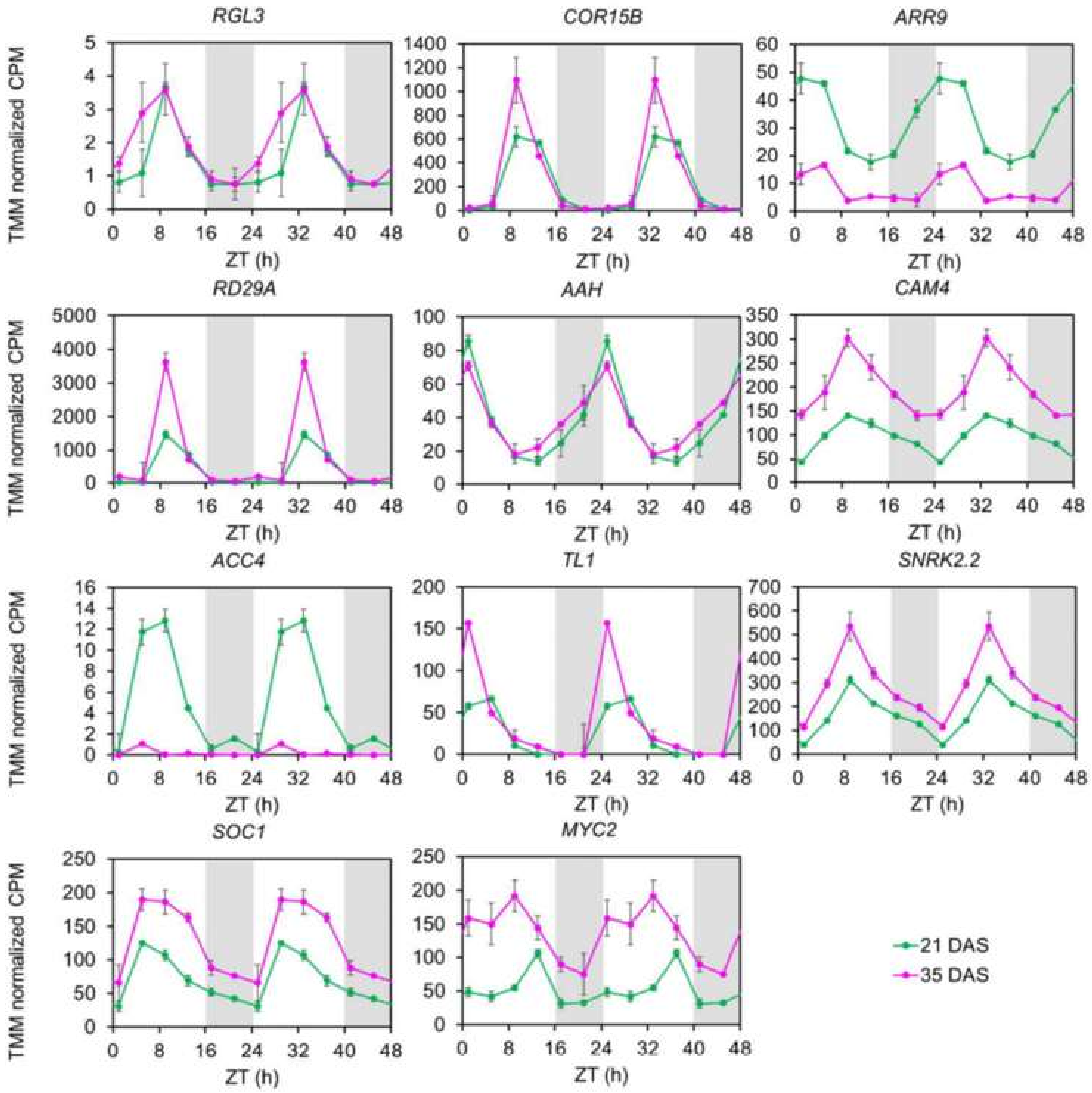

Supplementary Figure 5. Age-dependent changes in the patterns of daily oscillations in senescence regulators. The 24 h interval gene expressions were used duplicate for visualizing oscillation patterns **(A)**. Numbers of senescence regulators showing changes in the mean level of expression between 35 DAS plants and 21 DAS plants. The numbers of genes with no oscillation, young preferential single oscillation, old-preferential oscillation, and common oscillation are shown. **(B)** Examples of age-dependent changes in diurnal oscillation patterns of senescence regulators among common single oscillation genes with significant changes in the length of FWHM (p-value < 0.05 and |ΔFWHM| > 2h in the permutation test). The expression levels were normalized to show FWHM. Data are represented as mean ± SD. **(C)** The FWHMs of common single oscillation genes change between 21 DAS and 35 DAS. *RGL3*, *RD29A*, *ACC4*, *COR15B*, and *AAH* are negative regulators of senescence; *TL1*, *ARR9*, *CAM4*, *SNRK2.2*, *SOC1*, and *MYC2* are positive regulators of senescence in 21 and 35 DAS plants. Age-dependent change of the ascending and descending time was subjected to the permutation test (n = 1000; **p-value < 0.01 and *p-value < 0.05). Data are represented as mean ± SD.

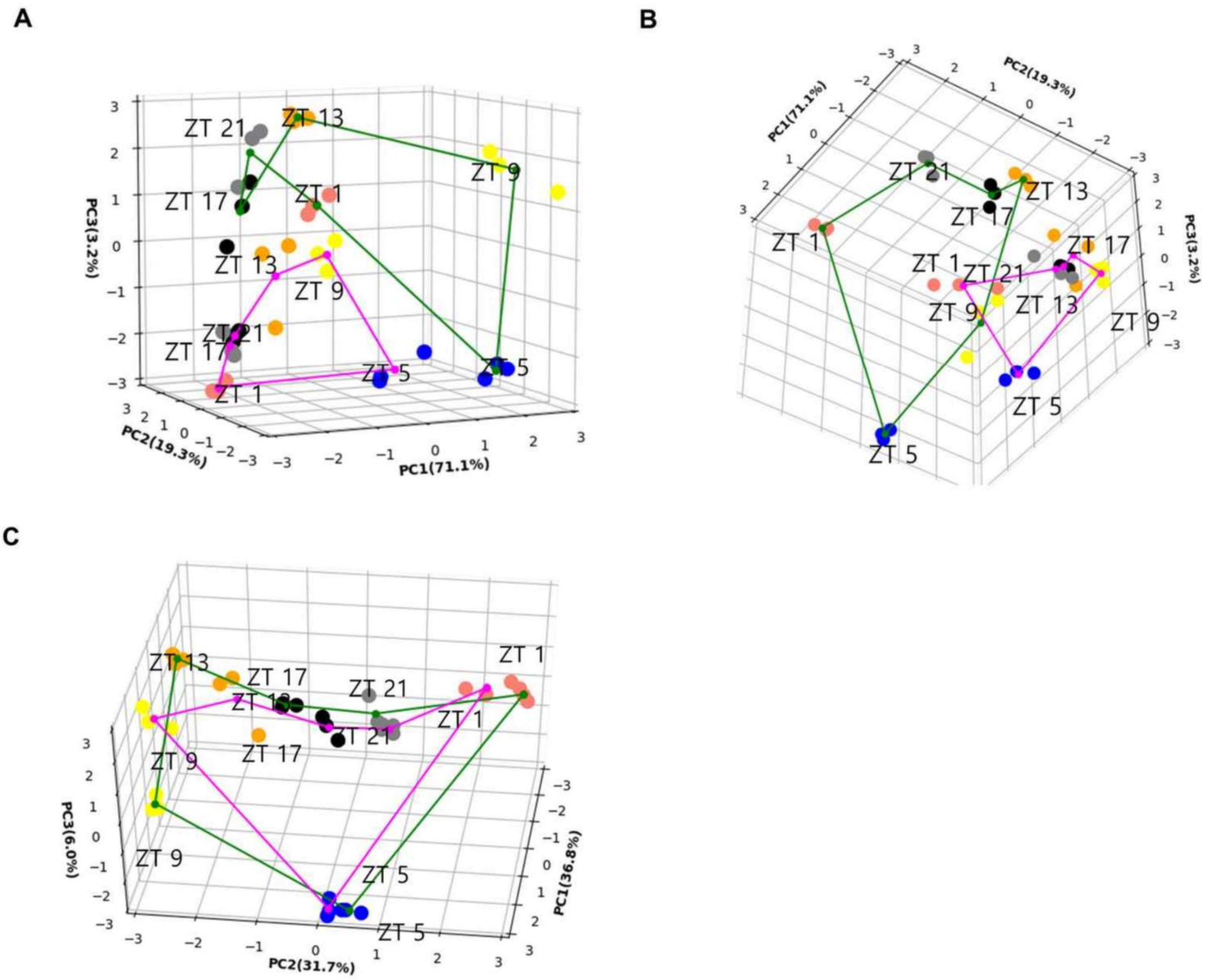

Supplementary Figure 6. Three-dimensional PCA analyses of the common SOGs at 21 and 35 DAS. **(A,B)** PCA analyses of the transcriptomes at 21 (green line) and 35 (magenta line) DAS. (A) and (B) are projections at different angles. **(C)** PCA analysis of the transcriptomes after normalization of amplitudes.

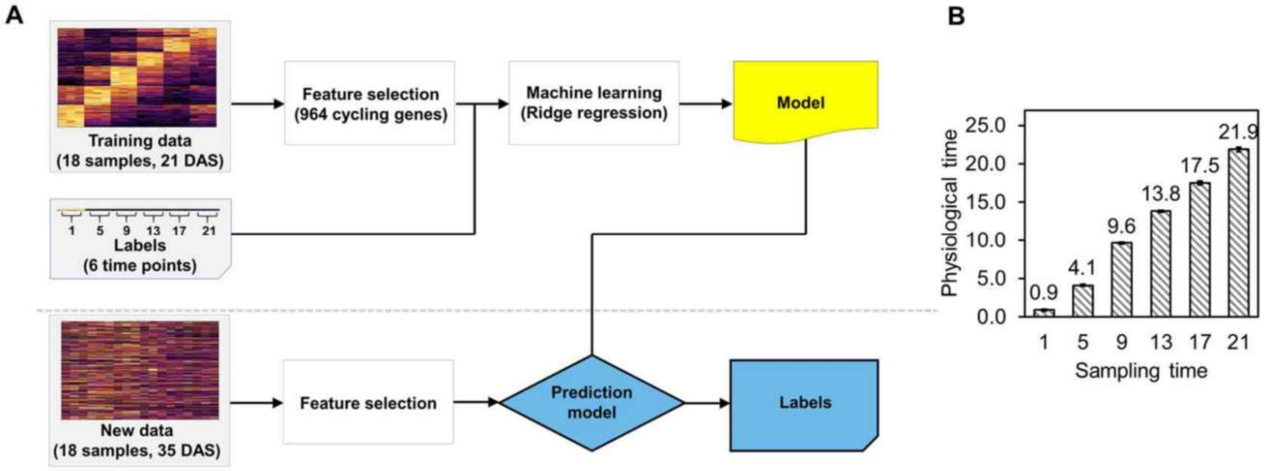

Supplementary Figure 7. Calculation and validation of subjective time. **(A)** Flow chart showing the calculation of subjective time from the transcriptomic data. Six time points, each with three replicates (a total of 18 data points), were used to create the model. The learning system based on ridge regression uses the expression values of the 960 common SOGs as input data and their sampling time as a label. The value predicted for the sampling time from the gene expression data given through the learned model is defined as subjective time. **(B)** Cross-validation analysis to validate the model's function and parameterization of subjective time of the transcriptomes. To verify the reliability of the subjective time produced by the model, we created a model based on data from which one of the samples (collected at ZT1, 5, 9, 13, 17, and 21) had been removed. We used the sample not utilized for training as an input to predict the sampling time. The predicted time showed less than 2 hours of errors compared to the actual sampling time.

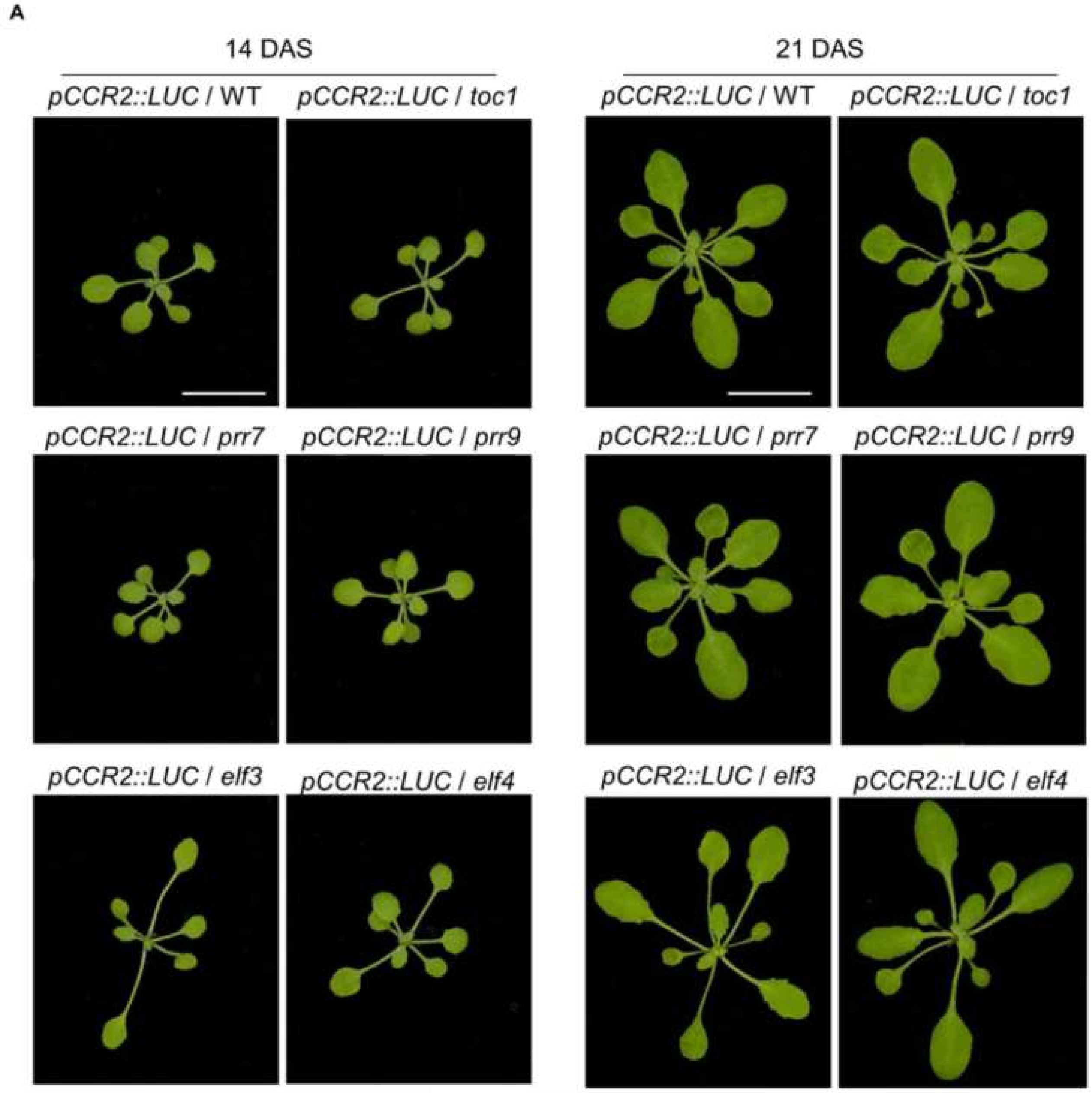

Supplementary Figure 8. Representative image of the plants on 14 and 21 DAS with Col-0 and mutations in core clock components. **(A,B)** Representative photos of plants used in the experiments for measuring luciferase activity in the 1st, 3rd, and 5th leaves in Col-0 and the mutant backgrounds at 14 DAS (A) and 21 DAS (B). Scale bar, 1 cm.

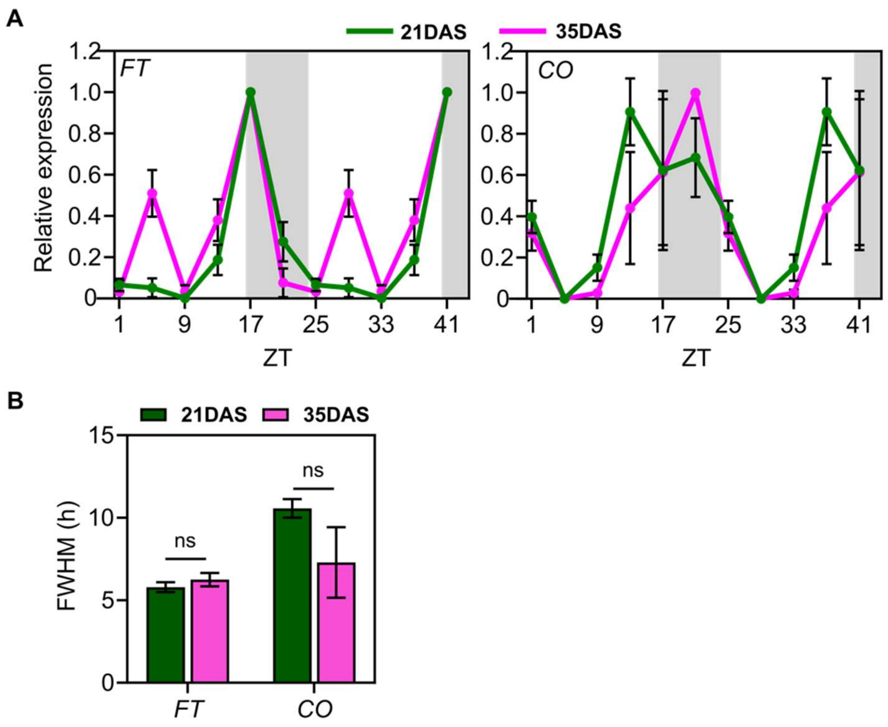

Supplementary Figure 9. Age-dependent circadian rhythms of representative flowering genes. (A) Diurnal expression patterns of *FT* and *CO* under long-day conditions. RNA-seq data were normalized using the Min-max normalization method to compare waveform and period. Expression profiles for each gene at different ages are represented by lines (21 DAS, green; 35 DAS, magenta). For visualization purposes, the expression data from ZT1 – ZT21 were duplicated and plotted from ZT25 – ZT41. (B) Age-dependent changes in the FWHM values of *FT* and *CO* mRNA expression. Statistical significance was evaluated using a two-tailed *t*-test (n = 3 biological replicates per condition). ns, no significant differences.
